# Job strain and resting heart rate: a cross-sectional study in a Swedish random working sample

**DOI:** 10.1186/s12889-016-2900-9

**Published:** 2016-03-05

**Authors:** Peter Eriksson, Linus Schiöler, Mia Söderberg, Annika Rosengren, Kjell Torén

**Affiliations:** Section of Occupational and Environmental Medicine, Institute of Medicine, Sahlgrenska Academy, University of Gothenburg, Box 414, S-405 30 Gothenburg, Sweden; Department of Molecular and Clinical Medicine, Su Sahlgrenska, 4135 45 Gothenburg, Sweden

**Keywords:** Work-related stress, Job strain, Job demands, Job control, Resting heart rate

## Abstract

**Background:**

Numerous studies have reported an association between stressing work conditions and cardiovascular disease. However, more evidence is needed, and the etiological mechanisms are unknown. Elevated resting heart rate has emerged as a possible risk factor for cardiovascular disease, but little is known about the relation to work-related stress. This study therefore investigated the association between job strain, job control, and job demands and resting heart rate.

**Methods:**

We conducted a cross-sectional survey of randomly selected men and women in Västra Götalandsregionen, Sweden (West county of Sweden) (*n* = 1552). Information about job strain, job demands, job control, heart rate and covariates was collected during the period 2001–2004 as part of the INTERGENE/ADONIX research project. Six different linear regression models were used with adjustments for gender, age, BMI, smoking, education, and physical activity in the fully adjusted model. Job strain was operationalized as the log-transformed ratio of job demands over job control in the statistical analyses.

**Results:**

No associations were seen between resting heart rate and job demands. Job strain was associated with elevated resting heart rate in the unadjusted model (linear regression coefficient 1.26, 95 % CI 0.14 to 2.38), but not in any of the extended models. Low job control was associated with elevated resting heart rate after adjustments for gender, age, BMI, and smoking (linear regression coefficient −0.18, 95 % CI −0.30 to −0.02). However, there were no significant associations in the fully adjusted model.

**Conclusions:**

Low job control and job strain, but not job demands, were associated with elevated resting heart rate. However, the observed associations were modest and may be explained by confounding effects.

## Background

There are several models to evaluate stressing work conditions. The most extensively studied is the job strain model developed by Karasek in 1979 [1]. This model postulates that the combination of high psychological demands and low decision latitude – that is, the combination of skill discretion and autonomy, often referred to as “control” – at work, results in high job strain and a risk of developing stress-related diseases [1, 2].

A variety of data indicate an association between job strain and an elevated risk of cardiovascular disease as a whole [3–7]. However, the evidence is ambiguous. One problem has been the lack of sufficient power in many studies [8]. There have also been great differences in study design, which has made it impossible to conduct meta-analyses in several systematic reviews [3, 4, 9], and there may be a problem with publication bias [6].

To support the existing data, several previous studies have investigated the association between job strain and established risk factors for cardiovascular disease. For example, job strain has been significantly associated with obesity [10, 11], physical inactivity, diabetes [10] and smoking [10, 12] in several large meta-analyses of cross-sectional data. Such associations make a relationship between job strain and cardiovascular risk biologically plausible. Additionally, they shed light on the etiologic mechanisms of this association.

We have chosen to study the association between job strain and resting heart rate. High resting heart rate or an elevation of heart rate over time has been associated with an increased risk of coronary heart disease death, cardiovascular death and/or all-cause mortality [13–17]. Three of the studies classified high resting heart rate as an independent risk factor [13, 15, 17]. The associations are strongest for the most extreme heart rates above 80–90 bpm, but increasing risks for heart rates above 65 bpm have also been described [16].

Little research has previously been published on the association between job strain and heart rate. To summarize the existing evidence, we performed a literature review in Medline and Scopus and found only a few studies. One retrospective cohort study of 500 male petrochemical industry workers, from 2013, reported a significant association between job strain and high resting heart rate [18]. The frequency of high heart rates ≥90 bpm was 10.2 % in the group with high job strain compared to 1.4 % in the group with low job strain (the variable high demands/low control was separated into three categories – high, medium, and low job strain).

A few smaller studies exploring the association between ambulatory heart rate, as an indicator of cardiovascular response, and job strain or the demand and control variables separately have shown varying results. Most of them have reported null findings [19–23], but one study actually reported a significantly higher baseline heart rate in groups with high strain compared to low strain, although it was not the main result [24]. Another study showed that low job control, but not the combination of low control and high psychological demands, is an important predictor of heart rate in men, not only during working hours but also during night time [25].

Hence, to date the evidence is sparse and inconclusive. We therefore conducted a population-based study with the following aims:

- **Specific aim:** To explore the association between job strain, job control, and psychological demands at work and resting heart rate in a random working sample, and to examine whether the association differs by gender.
- **General aim:** To give further basis for the epidemiologic evidence connecting work-related stress with an increased risk of cardiovascular disease and to investigate etiologic mechanisms of this association.
- **Hypothesis:** Job strain, high psychological demands, and low job control are positively associated with an elevated resting heart rate.

## Methods

### Population and data collection

In this cross-sectional study the subjects were recruited from the data register of the INTERGENE and ADONIX research projects. INTERGENE is a population-based study exploring the interplay between genetic susceptibility and environmental factors, life-style, and so forth, and the risk of cardiovascular diseases, in the general population in Västra Götalandsregionen, Sweden (West county of Sweden) [26]. ADONIX (Adult Onset Asthma and Nitric Oxide) is a subproject within this study, focusing on asthma [27]. The data collection was carried out from April 2001 until the end of 2004, and the study population consisted of randomly selected controls and coronary heart disease cases, and their first-degree relatives [28]. Only the randomly selected sample was included in our study.

General procedures in the studies were as follows: The participants were mailed study information, a questionnaire, and an invitation to a basic clinical examination where they also received complementary questionnaires containing questions on, among other things, the psychosocial situation at work [28]. Altogether, 8820 persons were invited, and of these, 8626 were eligible (194 were deceased, had an unknown address, or had moved abroad or to another part of Sweden) [29]. The response rate was 41.9 %, and the final sample consisted of 3614 men and women aged 25–74 years. All participants gave informed consent to be included. The studies were approved by the regional ethical review board at Gothenburg University (application numbers Ö092-91 for ADONIX and Ö237-2000 for INTERGENE). Detailed information about the INTERGENE and ADONIX research projects is available on the University of Gothenburg’s website [26].

Since our study demanded information on psychosocial work variables, all subjects not currently working full- or part-time (*n* = 1332) were excluded, leaving 2282 eligible subjects. Of these, 730 subjects could not be included in the statistical analyses due to missing information: 28 were lacking information on working status; 249 (of the remaining 2254 subjects) had missing (*n* = 246) or incomplete (*n* = 3) ECGs, that was used to measure resting heart rate; 379 (of the remaining 2005) did not fill in the psychosocial questionnaire and 74 (of the remaining 1626) had missing or incomplete information on demand and control variables. Hence, the final study population consisted of 1552 subjects, 752 men and 800 women.

### Variables

#### Resting heart rate

A standard 12-lead resting ECG was used to measure heart rate. The participants rested for 5 to 10 min before registering. A Siemens Megacart utilizing the Glasgow Royal Infirmary Interpretive ECG Algorithm was used to read the ECGs.

#### Psychosocial work variables and covariates

Information about psychosocial work variables and covariates was collected from the questionnaires filled in at home and at the clinical examination.

##### Demand and control

Demand and control were explored using a questionnaire with three Likert-scaled items (questions) for each variable. The questionnaire was based on a remodeled version [30, 31], of Karasek’s Job Content Questionnaire [32]. The variables of the structure describe similar dimensions as the job strain model, and will therefore be referred to as demand and control in this study. A sample demand item was; “How often during the last year has there been an increased amount of work?” and for control; “Do you have the possibility to decide your work tasks?”. Each item was scored 1 to 5 (answers ranging from “never” to “almost all the time”). A high score indicated high demand or high control respectively for the particular item. The scores were summed, giving a discrete value between 3 and 15 for each dimension. The median score was 10 for demands and 11 for control. We used the log-transformed ratio of job demands over job control to operationalize job strain in the statistical analyses. We also analyzed the sum scores of the control and demand variables separately. Internal consistency was calculated using Cronbach’s alpha and was good for job control (0.81) and acceptable for job demands (0.76).

#### Statistical methods

Statistical calculations were performed with SAS, version 9.4 for Windows (SAS institute; Cary; NC). Linear regression analyses were performed with resting heart rate as dependent variable. Resting heart rate was used as a continuous variable in all analyses. Job strain, operationalized as the log-transformed ratio of the sum scores of job demands over job control $\left( \ln \left(\frac{\mathrm{job}\ \mathrm{demands}}{\mathrm{job}\ \mathrm{control}}\right)\right)$, “Job control,” and “Job demands” were used as continuous variables. A previous study of alternative formulations of job strain has shown that the log-transformed ratio is a good measure of job strain and the best predictor of stress, compared to the quadrant, quotient and subtraction approach [33]. Also, log-transformation was necessary since the ratio of job demands over job control was very skewed and not appropriate to include in the linear regression models. For each variable, one model was unadjusted and one model was adjusted for gender and age. Then we successively added BMI, smoking, education, and physical activity, generating six models in total, with adjustments for gender, age, BMI, smoking, education, and physical activity in the fully adjusted model. Age and BMI were used as continuous variables and gender as a dichotomous variable, with “female” as reference category. Smoking, physical activity, and education were all self-reported categorical variables. The smoking variable comprised the categories “never smoker,” “ex-smoker,” and “current smoker,” and was converted to dummy variables with “never smoker” as reference category. Physical activity and education were both dichotomized into two categories: “Regular exercise and training during leisure time”, and “hard exercise or competitive sports,” were classified as high, and “sedentary leisure time” and “moderate exercise during leisure time,” were classified as low physical activity (reference). Corresponding categories for education was “university education” (high), and “primary school,” “lower secondary school,” and “upper secondary school” (low, reference). Education was considered to be a marker of socioeconomic status.

Further, we present general characteristics of the study sample in relation to gender, resting heart rate, and the non-transformed ratio of job demands over job control (referred to as the “job strain-ratio”). The ratio was dichotomized into two categories: “job strain-ratio >1” and “job strain-ratio ≤1”. We chose “1” as a natural cut-off since it divides the population into those who reported a higher job demand-score than job control-score (job strain-ratio >1) and those who reported a higher job control-score than job demand-score (or equal scores – “job strain-ratio ≤1”). This cut-off has also been used in previous studies [34].

The Spearman correlation was calculated between resting heart rate and the log-transformed job strain-ratio, “job control,” and “job demands,” respectively, and internal consistency for the demand and control measures was calculated using Cronbach’s alpha. We also performed interaction tests by gender for the exposure variables “job strain,” “job demands,” and “job control”. All tests were double sided and a *p*-value <0.05 was considered significant.

## Results

Table 1 shows general characteristics of the study population. In total, 621 of 1552 participants (40.0 %) had a job strain-ratio >1. A job strain-ratio >1 was more common among women (46.9 %) than men (32.7 %) (*p*-value <0.01). Further, women had a higher mean resting heart rate (62.4 bpm compared to 59.4 bpm, *p*-value <0.01) and were more likely to smoke than men. Men were slightly older (mean age 46.6 years compared to 45.4 years) and had higher blood pressure, mean BMI, and waist measure than women. Additionally, there were significant overall associations between gender and BMI, education, and physical activity respectively.

**Table 1:** General characteristics of the study sample

	All	Men	Women	*p*-value^*^
Total, *n* (%)^a^	1552	752 (48.5)	800 (51.5)	
-Job strain ratio >1, *n* (%)^b^	621 (40.0)	246 (32.7)	375 (46.9)	<0.01
-Job strain ratio ≤1, *n* (%)^b^	931 (60.0)	506 (67.3)	425 (53.1)	
Mean age	46.0	46.6	45.4	0.02
Age, range (median)	24–71 (46)	24–71 (47)	25–68 (45)	
Mean heart rate (SD)	61.0 (9.9)	59.4 (10.0)	62.4 (9.5)	<0.01
Heart rate, range (median)	35–109 (60)	35–109 (59)	39–101 (62)	
Mean diastolic blood pressure (mmHg)	81.3	82.5	80.2	<0.01
Mean systolic blood pressure (mmHg)	126.6	130.5	122.9	<0.01
Mean BMI (kg/m^2^)	25.5	26.2	24.8	<0.01
BMI, *n* (%)^b^				<0.01^**^
-Underweight (BMI <18.5 kg/m^2^)	8 (0.5)	0 (0)	8 (1.0)	
-Normal weight (18.5 ≤BMI <25 kg/m^2^)	769 (49.5)	300 (39.9)	469 (58.6)	
-Overweight (25 ≤BMI <30 kg/m^2^)	602 (38.8)	355 (47.2)	247 (30.9)	
-Obese (BMI ≥30 kg/m^2^)	173 (11.1)	97 (12.9)	76 (9.5)	
Waist measure, mean (cm)	86.5	92.9	80.2	<0.01
Current smokers, %	260 (16.9)	95 (12.8)	165 (20.7)	<0.01
Leisure time physical activity, *n* (%)^b^				<0.01
-Sedentary leisure time	147 (9.5)	86 (11.5)	61 (7.6)	
-Moderate exercise during leisure time	889 (57.4)	409 (54.5)	480 (60.1)	
-Regular exercise and training	468 (30.2)	225 (30.0)	243 (30.4)	
-Hard exercise or competitive sports	45 (2.9)	30 (4.0)	15 (1.9)	
Education, *n* (%)^b^				<0.01
-Compulsory school	193 (12.5)	119 (15.8)	74 (9.3)	
-Lower secondary school	336 (21.7)	165 (21.9)	171 (21.5)	
-Upper secondary school	424 (27.4)	219 (29.1)	205 (25.7)	
-University education	596 (38.5)	249 (33.1)	347 (43.5)	

^a^Row percentage

^b^Column percentage

^*^Presented for significant differences between men and women. T-test was used for continuous variables and chi2 for categorical variables

^**^Fisher’s exact test was used since the number of underweight subjects in one of the categories were less than 5, rendering the chi2-test potentially invalid

General characteristics in relation to the job strain-ratio are presented in Table 2. People presenting a job strain-ratio >1 had a higher mean resting heart rate (*p*-value 0.03) and were more likely to smoke than people presenting a job strain-ratio ≤1 (*p*-value 0.01). They also exhibited a smaller waist measure. Physical activity, but not BMI and education, was significantly associated with the dichotomized job strain-ratio. Lastly, characteristics in relation to resting heart rate are presented in Table 3. A resting heart rate ≤25^th^ percentile (54 bpm) was classified as low, and ≥75^th^ percentile (67 bpm) as high. Subjects with high resting heart rate were older, more likely to be women, and more likely to present a job strain-ratio >1, than subjects with low resting heart rate (*p*-value <0.01 in all cases). Furthermore, there was a clear trend with increasing blood pressure, BMI, and waist measure with increasing resting heart rate. Subjects with high resting heart rate were also more likely to smoke and there were significant overall associations between high and low resting heart rate and BMI, exercise, and education respectively.

**Table 2:** General characteristics in relation to job strain

	Job strain-ratio >1	Job strain-ratio ≤1	*p*-value^*^
Total, *n* (%)^a^	621 (40.0)	931 (60.0)	
Mean age	45.5	46.3	ns
Age, range (median)	25–64 (46)	24–71 (46)	
Mean heart rate (SD)	61.6 (9.5)	60.5 (10.1)	0.03
Heart rate, range (median)	38*–*92 (61)	35–109 (60)	
Mean diastolic blood pressure (mmHg)	81.5	81.2	ns
Mean systolic blood pressure (mmHg)	125.8	127.1	ns
Mean BMI (kg/m^2^)	25.4	25.5	ns
BMI, (%)^b^			ns^**^
-Underweight (BMI <18.5 kg/m^2^)	4 (0.6)	4 (0.4)	
-Normal weight (18.5 ≤BMI <25 kg/m^2^)	308 (49.6)	461 (49.5)	
-Overweight (25 ≤BMI <30 kg/m^2^)	239 (38.5)	363 (39.0)	
-Obese (BMI ≥30 kg/m^2^)	70 (11.3)	103 (11.1)	
Waist measure, mean (cm)	85.6	87.1	0.02
Current smokers, *n* (%)	125 (20.4)	135 (14.6)	<0.01
Leisure time physical activity, n (%)^b^			0.02
-Sedentary leisure time	68 (11.0)	79 (8.5)	
-Moderate exercise during leisure time	375 (60.4)	514 (55.4)	
-Regular exercise and training	164 (26.4)	304 (32.8)	
-Hard exercise or competitive sports	14 (2.3)	31 (3.3)	
Education, *n* (%)^b^			ns
-Compulsory school	83 (13.6)	110 (12.0)	
-Lower secondary school	134 (20.6)	202 (22.1)	
-Upper secondary school	177 (28.1)	247 (27.1)	
-University education	226 (37.7)	370 (38.8)	

^a^Row percentage

^b^Column percentage

^*^Presented for significant differences between “job strain-ratio >1” and “job strain-ratio ≤1”. T-test was used for continuous variables and chi2 for categorical variables

^**^Fisher’s exact test was used since the number of underweight subjects in the different categories were less than 5, rendering the chi2-test potentially invalid

**Table 3:** General characteristics in relation to resting heart rate^a^

	HF ≤25^th^ percentile	25^th^ <HF ≤50^th^ percentile	50^th^ <HF <75^th^ percentile	HF ≥75^th^ percentile	*p*-value^*^
Total, *n* (%)^b^	409 (26.4)	384 (24.7)	366 (23.6)	393 (25.3)	
-Men, *n* (%)^c^	248 (60.6)	190 (49.5)	162 (44.3)	152 (38.7)	<0.01
-Women, *n* (%)^c^	161 (39.4)	194 (50.5)	204 (55.7)	241 (61.3)	
-Job strain ratio >1, *n* (%)^c^	138 (33.7)	160 (41.7)	151 (41.3)	172 (43.8)	<0.01
-Job strain ratio ≤1, *n* (%)^c^	271 (66.3)	224 (58.3)	215 (58.7)	221 (56.2)	
Mean age	44.4	46.7	46.3	46.6	<0.01
Age, range (median)	25–69 (44)	26–67 (48)	25–66 (47)	24–71 (48)	
Mean diastolic blood pressure (mmHg)	78.5	80.8	82.6	83.7	<0.01
Mean systolic blood pressure (mmHg)	123.4	126.1	127.5	129.5	<0.01
Mean BMI (kg/m^2^)	24.8	25.3	25.6	26.3	<0.01
BMI, n (%)^c^					<0.01^**^
-Underweight (BMI <18.5 kg/m^2^)	1 (0.2)	1 (0.3)	3 (0.8)	3 (0.8)	
-Normal weight (18.5 ≤BMI <25 kg/m^2^)	235 (57.5)	198 (51.6)	167 (45.6)	169 (43.0)	
-Overweight (25 ≤BMI <30 kg/m^2^)	152 (37.2)	147 (38.3)	153 (41.8)	150 (38.2)	
-Obese (BMI ≥30 kg/m^2^)	21 (5.1)	38 (9.9)	43 (11.8)	71 (18.1)	
Waist measure, mean (cm)	84.9	86.2	87.0	88.1	<0.01
Current smokers, (%)^c^	54 (20.8)	61 (23.5)	74 (28.5)	71 (27.3)	0.05
Leisure time physical activity, *n* (%)^c^					<0.01
-Sedentary leisure time	25 (6.1)	39 (10.2)	39 (10.7)	44 (11.2)	
-Moderate exercise during leisure time	175 (42.9)	225 (58.8)	222 (60.7)	267 (68.1)	
-Regular exercise and training	182 (44.6)	111 (28.9)	97 (26.5)	78 (19.9)	
-Hard exercise or competitive sports	26 (6.4)	8 (2.1)	8 (2.2)	3 (0.8)	
Education, *n* (%)^c^					<0.01
-Compulsory school	39 (9.5)	42 (10.9)	55 (15.1)	57 (14.5)	
-Lower secondary school	71 (17.4)	84 (21.9)	80 (22.0)	101 (25.7)	
-Upper secondary school	119 (29.1)	103 (26.8)	95 (26.1)	107 (27.4)	
-University education	180 (44.0)	155 (40.4)	134 (36.8)	127 (32.4)	

^a^25^th^ percentile = 54 bpm, 50^th^ percentile = 60 bpm, 75^th^ percentile = 67 bpm

^b^Row percentage

^c^Column percentage

^*^Presented for significant differences between HF ≤25^th^ percentile and HF ≥75^th^ percentile. *T*-test was used for continuous variables and chi2 for categorical variables

^**^Fisher’s exact test was used since the number of underweight subjects in the different categories were less than 5, rendering the chi2-test potentially invalid

The Spearman correlation between resting heart rate and the log-transformed job strain-ratio was 0.058, which was statistically significant (*p*-value 0.02). For job control, the Spearman Correlation was −0.075 (*p*-value 0.003), while the correlation with job demands was weaker and non-significant (−0.016, *p*-value 0.53).

Linear regression analyses did not display any significant association between job demands and resting heart rate (Table 4). Job strain was significantly associated with resting heart rate in the unadjusted model (regression coefficient 1.26, 95 % CI 0.14 to 2.38, *p*-value 0.03). That is, increasing job strain was associated with increasing heart rate. However, successively adding gender and age, BMI, smoking, education, and exercise to the model, gradually attenuated the association, which was weak in the fully adjusted model (regression coefficient 0.37, 95 % CI −0.72 to 1.46, *p*-value 0.51). The significance disappeared already in model 2, adjusted for gender and age (although the association was close to significant here), but the greatest attenuation was seen when exercise was added to the model in the last step.

**Table 4:** Linear regression between job strain and resting heart rate

	Job strain (95 % CI)	*p*-value	Job demands (95 % CI)	*p*-value	Job control (95 % CI)	*p*-value
Model 1	**1.26 (0.14;2.38)**	**0.03**	−0.15 (−0.37;0.06)	0.17	**−0.26 (−0.42;−0.10)**	**0.001**
Model 2	1.09 (−0.02;2.21)	0.05	−0.13 (−0.35;0.08)	0.22	**−0.21 (−0.36;−0.05)**	**0.01**
Model 3	0.83 (−0.27;1.94)	0.14	−0.17 (−0.38;0.04)	0.12	**−0.19 (−0.34;−0.03)**	**0.02**
Model 4	0.75 (−0.35;1.86)	0.18	−0.17 (−0.38;0.03)	0.10	**−0.18 (−0.33;−0.02)**	**0.02**
Model 5	0.59 (−0.52;1.70)	0.30	−0.14 (−0.35;0.07)	0.19	−0.14 (−0.30;0.01)	0.08
Model 6	0.37 (−0.72;1.46)	0.51	−0.16 (−0.36;0.05)	0.14	−0.10 (−0.26;0.05)	0.18

Significant results are in bold. Intercepts are not presented

Model 1: Unadjusted

Model 2: Adjustments were made for gender and age

Model 3: As model 2 but further adjustments were made for BMI

Model 4: As model 3 but further adjustments were made for smoking

Model 5: As model 4 but further adjustments were made for education

Model 6: As model 5 but further adjustments were made for leisure time physical activity

Likewise, job control was significantly associated with resting heart rate in the unadjusted model (regression coefficient −0.26, 95 % CI −0.42 to −0.10, *p*-value 0.001) (Table 4). That is, persons perceiving low control presented higher resting heart rate than those who did not. Again, the association gradually attenuated for each new variable entered to the model, but it was still significant after adjustments for gender, age, BMI, and smoking (regression coefficient −0.18, 95 % CI −0.30 to −0.02, *p*-value 0.02). However, additional adjustments for education and exercise, attenuated the association to the null (regression coefficient −0.10, 95 % CI −0.26 to 0.05, *p*-value 0.18, in the fully adjusted model). Two way interactions between gender and job strain, job demands, and job control respectively, were also added to the different models. None of the interaction effects reached statistical significance.

## Discussion

In this cross-sectional study, there was no association between job demands and resting heart rate. Job strain was associated with elevated resting heart rate in an unadjusted linear regression model, but not in any of the extended models. Low job control was associated with elevated resting heart rate after adjustments for gender, age, BMI, and smoking, but the association was modest and the significance disappeared after additional adjustment for education and leisure time physical activity. Several previous studies have investigated the association between ambulatory heart rate, as an indicator of cardiovascular response, and job strain or the demand and control variables separately [19–24]. The majority reported null findings [19–23]. Thus, the present study supports previous observations of no relation between heart rate and the demand and control variables, although the outcome in our study was slightly different.

Yet, a few studies have shown conflicting results. One study reported a higher baseline heart rate in groups with high strain compared to low strain, although it was not the main result [24]. Another study used ambulatory measurements to show that low perceived job control (that is, the perception of control at a given moment), but not job strain, predicted heart rate in men (*n* = 149) during working hours and night time [25], and one retrospective cohort study of 500 male petrochemical industry workers reported a significant association between job strain and high resting heart rates ≥90 bpm [18]. It is of course possible that we have underestimated the effect of work-related stress on resting heart rate. One reason for this may be that we used a questionnaire based on a remodeled version of Karasek’s job demand-control structure, which possibly did not capture the dimensions of the model properly. Another reason may be the cross-sectional design of this study. Due to this, we do not know anything about the time of exposure to high demands and low control. Since cardiac disease develops over a long period of time, it is possible that we have underestimated the risk of elevated heart rate, if a section of the participants reporting job strain have only been exposed for a shorter time.

Additionally, although the association between job strain and low job control, and resting heart rate, probably can be explained by confounding effects, another possibility is that one or more of the variables used as covariates actually are intermediate factors. Smoking [10, 12] and obesity [10, 11] has been significantly associated with job strain in previous studies. You could hypothesize that stressing work conditions increases the risk for an un-healthy lifestyle. However, even though smoking was associated with both resting heart rate and a job strain-ratio >1 in the present study, adding smoking to the model had minimal effect on the association and hence, smoking probably plays a minor role.

Further, it is not unlikely that education, as a marker of socioeconomic status, is an effect modifier, so that low education and low control have multiplicative effects. To explore this, two way interactions between education and job strain, job demands, and job control respectively, were added to the different models (results not shown), but as for gender, none of the interaction effects reached statistical significance**.** Furthermore, physical inactivity may be a possible mediator in the relationship between job control and elevated resting heart rate. In our study, the job strain population had a tendency to exercise less (see Table 2) and physical inactivity has been significantly associated with job strain in previous studies [10]. In conclusion, smoking seemed to play a minor role in the relationship between resting heart rate and stressing work conditions, and education was not an effect modifier in the present study, but obesity may act as an intermediate factor and physical inactivity as a mediator. Thus, it can be speculated that the more extended statistical model entails a risk of overcompensation that can hide a true association.

An actual relationship would be interesting from a pathophysiological perspective. As previously mentioned, an elevated resting heart rate has been associated with increased cardiovascular risk in several studies [13–16]. Further, it is known that resting heart rate is highly dependent on the inflow of sympathetic and parasympathetic activity to the heart. High resting heart rate has accordingly also been shown to be associated with increased sympathetic activity [35]. Autonomic imbalance in terms of increased sympathetic and/or decreased vagal activity has been related to a number of conditions, including cardiovascular disease [36]. Such an autonomic imbalance – visible as decreased heart rate variability – has been associated with job strain, although the evidence is somewhat contradictory [37]. Hence, if there in fact is a relationship between elevated heart rate and job strain or low job control, it would provide some evidence that autonomic imbalance is the pathophysiological link between work-related stress and cardiovascular disease. This would also be in accordance with the hypotheses made by the job strain model. It is essential to stress, though, that the effects of job strain and job control on resting heart rate were modest and the correlations low. The association might therefore be of limited importance.

### Methodological strengths and limitations

The most important advantage of this study is that we investigated a relatively large random sample in a general population. This increases external validity. The limitations include the cross-sectional design that, apart from what is previously mentioned, does not give any information about the chronology of exposure and outcome: that is, conclusions about causality cannot be made. Another limitation was that the evaluation of psychosocial work characteristics was based on a remodeled version of Karasek’s Job-demand structure [32, 38]. Although the theoretical content was the same and internal consistency was good, dimensional validity measures were lacking. Therefore, some caution must be taken when interpreting the results since misclassification of the dimensions of the model might introduce bias towards the null.

Furthermore, there was a relatively large loss of information (*n* = 730) that entails reduced power and a risk of inadequate precision. Additionally, since the response rate in the INTERGENE and ADONIX studies was only 41.9 %, there is a risk that the composition of the study sample might be skewed due to selection bias. A previous study [29] showed that people who declined the invitation to attend in the INTERGENE and ADONIX studies were more likely to be young, men, have lower education, and originate from outside Scandinavia. The selective drop-out of young men may have led to an overestimation of the risk of elevated resting heart rate in men perceiving job strain or low job control, since the overall cardiovascular risk generally is lower in younger people. Also, the less frequent participation of subjects with lower education may have induced underestimation of the importance of low socioeconomic status.

### Suggestions for future studies

To increase external validity, future studies would take advantage of using the Job Content Questionnaire when exploring psychosocial work characteristics.

## Conclusions

Using cross-sectional data from a random sample of men and women, we present no association between job demands and resting heart rate. There were modest, but significant, associations between low job control and job strain, operationalized as the log-transformed ratio of job demands over job control, and resting heart rate. However, the associations may be explained by confounding effects. Since this was one of the first studies on this topic, more studies are needed to further explore the relationship between the demand and control variables, and resting heart rate.
